# Application of single-cell RNA sequencing methods to develop B cell targeted treatments for autoimmunity

**DOI:** 10.3389/fimmu.2023.1103690

**Published:** 2023-07-14

**Authors:** Catherine A. Nicholas, Mia J. Smith

**Affiliations:** ^1^ Barbara Davis Center for Diabetes, University of Colorado School of Medicine, Aurora, CO, United States; ^2^ Department of Biochemistry and Molecular Genetics, University of Colorado School of Medicine, Aurora, CO, United States; ^3^ Department of Pediatrics, University of Colorado School of Medicine, Aurora, CO, United States; ^4^ Department of Immunology and Microbiology, University of Colorado School of Medicine, Aurora, CO, United States

**Keywords:** scRNA-seq, B cells, autoimmunity, antigen-specific, autoreactive

## Abstract

The COVID-19 pandemic coincided with several transformative advances in single-cell analysis. These new methods along with decades of research and trials with antibody therapeutics and RNA based technologies allowed for highly effective vaccines and treatments to be produced at astonishing speeds. While these tools were initially focused on models of infection, they also show promise in an autoimmune setting. Self-reactive B cells play important roles as antigen-presenting cells and cytokine and autoantibody producers for many autoimmune diseases. Yet, current therapies to target autoreactive B cells deplete all B cells irrespective of their pathogenicity. Development of self-reactive B cell targeting therapies that would spare non-pathogenic B cells are needed to treat disease while allowing effective immune responses to other ailments. Single-cell RNA sequencing (scRNA-seq) approaches will aid in identification of the pathogenic self-reactive B cells operative in autoimmunity and help with development of more favorable precision targeted therapies.

## Introduction

B cells play a variety of roles in the pathogenesis and maintenance of autoimmunity, though their precise roles may differ between autoimmune disorders. Antibody production is one of the main hallmarks of B cell effector activity. When an autoreactive B cell produces self-reactive antibodies, these autoantibodies can lead to excessive inflammation and tissue damage through a variety of pathways, including immune-complex deposition, complement activation, Fc- receptor mediated antigen-antibody uptake by antigen-presenting cells (APCs) leading to activation of T cells, and Fc- receptor mediated activation of NK cells and APCs (1, 2). Autoimmune disorders such as systemic lupus erythematosus (SLE), pemphigus vulgaris, and Graves’ disease (GD) are known to be influenced by the presence of self-reactive antibodies. However, other autoimmune diseases are not as obviously impacted by autoantibodies, even if they are present, such as with type 1 diabetes (T1D), multiple sclerosis (MS), and Hashimoto’s thyroiditis. Additional effector roles of B cells that can contribute to autoimmune conditions include their ability to act as potent APCs to autoreactive CD4 and CD8 T cells and their ability to produce pro-inflammatory cytokines to stimulate other immune cells to act against the body (3). Despite the role of B cells in development of autoimmunity, a greater understanding of the specific pathogenic B cell subsets and their function are needed to develop more precise therapies. Here we provide an overview of how the use of single-cell RNA sequencing (scRNA-seq) to study rare B cell subsets can inform our understanding of the role of autoreactive B cells in development of autoimmunity and help guide future therapeutics targeting pathogenic B cells to treat, prevent, or delay autoimmunity. In addition, we discuss the benefits of scRNA-seq over existing high dimensional technologies, how scRNA-seq has already begun to be applied to the study of B cells in autoimmunity, and limitations of using scRNA-seq to study such rare B cells. We discuss some of the inconsistencies in the field that have arisen in the analysis of these cells, as well as the importance of considering the affinity of the B cell and the timing of blood or tissue collection.

### Targeting B cells for treatment of autoimmunity

In the past few decades, depletion of total B cells has mainly been driven by treatment with anti-CD20 monoclonal antibody therapy, such as rituximab or more recently, ocrelizumab. Initially developed to treat B cell cancers, such as chronic lymphocytic leukemia (CLL) and non-Hodgkin’s lymphoma (NHL), these therapies have also now been tested in various autoimmune disorders (4, 5). A phase 2 clinical trial using rituximab in new onset T1D patients demonstrated some efficacy with reduced requirement for insulin and preserved beta cell function one year after treatment. However, when the B cell compartment recovered post-treatment, autoreactive B cells returned and the disease progressed (6, 7). The ability of rituximab to deplete pathogenic B cells has been tested in other autoimmune conditions, including rheumatoid arthritis (RA) (8), Sjögren’s syndrome (9), myasthenia gravis (MG) (10), and SLE (11) culminating in variable success and not always resulting in longstanding benefits. One criticism of anti-CD20 B cell targeting therapies is that these drugs deplete all B cells irrespective of their antigen specificity, eliminating both pathogenic and non-pathogenic B cells. In addition, CD20 is not expressed on the surface of long-lived plasma cells, and therefore, anti-CD20 therapy does not readily deplete self-reactive autoantibody producing cells residing in the bone marrow, which would be important in autoantibody dependent autoimmune disorders, such as MG. Despite these concerns, anti-CD20 therapy is particularly effective in some autoimmune disorders, such as RA, demonstrating the importance of the other effector functions of B cells, including their ability to act as potent APCs to T cells.

Despite the demonstrated clinical benefits of global B cell depletion, the field is currently looking to target disease-related B cells rather than the entire B cell compartment. In particular, an improved understanding of which B cell subsets are contributing to disease (e.g. memory, plasma cell, atypical memory, germinal center (GC), extrafollicular) will help in targeting pathogenic cells with therapeutic treatments. To identify B cell subsets participating in autoimmune disease, a deeper understanding of the relevant antigen-specificities, clonotype, and both mRNA and protein expression phenotypes are required due to the heterogeneous landscape of autoimmune disease phenotypes and responses to treatment strategies. Traditional methods for obtaining these types of data are complicated and time consuming, frequently yielding results for only one readout with limitations on the depth of data collected. The ability of scRNA-seq to deeply phenotype antigen-specific B cells can now be applied to studies of B cells in the context of autoimmunity, which will help inform future precision based therapeutic targets.

### The advent of single-cell analysis of immune cells by sequencing

Flow cytometry has served for decades as an essential tool to phenotype and classify immune cells according to their expressed surface proteins, as well as cytosolic protein expression and activation state by phosphorylation status. The innovation of conjugating a detection antibody to a fluorophore and analyzing each cell in a sample for the intensity of emission detected has allowed immunologists to vastly expand our understanding of different cell types. While traditional flow cytometry has been limited by the number of markers that can be simultaneously analyzed to 10 or so due to issues with compensation, more recent advances have been made in spectral flow cytometry and mass cytometry (CyTOF) to expand panels to include ~30-50 targets per cell. The increase in the ability to analyze more markers in one panel has enabled researchers to more deeply phenotype a single immune cell type, as well as compare and correlate phenotypes across multiple cell lineages (i.e. B cells, T cells, myeloid cells) in a single sample. Using these approaches, new immunological biomarkers with complex phenotypes have been observed in patients suffering from autoimmune disorders. One notable example includes the identification of an expansion of T-bet expressing age-associated B cells (ABCs) (12) in various autoimmune disorders, which have been shown through the use of high parameter studies to have a heterogeneous phenotype that can vary depending on the autoimmune disease, stage of disease (newly diagnosed vs long-standing), age of patients (pediatric vs adult), and localization (tissue vs peripheral blood) (13–17).

Despite the recent advances in flow and mass cytometry to more deeply phenotype immune cells during development of autoimmunity, these methods are often limited to protein expression only, are unable to dive deeper into the transcriptome of cells of interest, informing cellular states of differentiation and activation, or provide information on unique aspects of B and T cell receptors. Further, these techniques are inherently biased, in that, researchers choose which markers to study *a priori* rather than discovering unique marker expression patterns in an unbiased manner. Sequencing technologies have revolutionized these types of data collection over the past several decades. In particular, the advent of RNA sequencing (RNA-seq) has allowed scientists to visualize transcriptome activity. Bulk RNA-seq identifies the current pre-translational gene expression state of a sample at the time of collection. However, when done in bulk, the signals are averaged across all cells in the sample, obscuring potential rare cell populations that may have different expression signals. In addition, identification of paired heavy and light chain sequencies are not achievable with bulk RNA-sequencing, impairing the ability to study the B cell receptor (BCR) acquired mutations, and antigen binding and affinity properties.

In the last ten years, this technology has developed from capturing RNA from a multicellular sample to the ability to measure mRNA expression of immune cells at the single-cell level. The various scRNA-seq technologies and their application to immunology have been nicely and extensively reviewed by others (18–20). In its simplest form, droplet-based single-cell capture and subsequent RNA sequencing enables analysis of the transcriptomic state of each individual cell in a sample by analysis of polyadenylated RNA transcripts, increasing the ability of researchers to identify unique cell populations of interest from a larger, mixed sample. Rather than pre-identifying genes of interest and assessing their mRNA expression levels by RT-qPCR, RNA sequencing captures the total mRNA expression of a cell at the time of collection, which has enabled researchers to identify new and more complete cell transcriptome phenotypes. Single-cell transcriptome measurements are highly valuable for many reasons; two of the most important reasons include: 1) they differentiate rare signals from cells within a larger tissue sample, particularly if the sample is heterogeneous, and 2) individual cell identities and differentiation states can be ascertained, often to seek out a particular cell of interest. The application of these methods is far reaching, and immunologists are making use of this tool to identify unique cell populations from scRNA-seq. Advancements with scRNA-seq methods now allow researchers to identify B cell Receptor (BCR) and T Cell Receptor (TCR) V(D)J sequences, observe somatic hypermutation (SHM), and identify clonal expansions within a sample. Add-on methods to scRNA-seq platforms can increase the data output of these experiments through the identification of surface proteins with oligo-tagged antibodies (CITE-seq) (21), analysis of the epigenome (ATAC-seq) (22), spatial distribution of cells within tissues (spatial transcriptomics) (23), and pre-sorting of antigen-specific cells prior to single-cell capture and sequencing (LIBRA-seq, discussed below)) (24), among other types of readouts. Until the advent of single-cell sequencing technology, this amount of granular data was not available on a per-cell and per-sample level. Hence, by combining these various applications of scRNA-seq, the research community has the potential to fill knowledge gaps regarding the phenotype (both protein and transcriptional) of autoreactive B cells, their differentiation state (25, 26), cellular trajectory (27, 28), mechanism of action, clonal expansion (29), location within tissues (30, 31), epigenetic changes (32), and interaction with other cell types during development of autoimmunity in an unbiased manner. With this knowledge comes the ability to identify more precise targets for the treatment or prevention of autoimmunity and a better understanding of the pathogenesis of autoimmune diseases in general.

### Baiting antigen-specific B cells prior to scRNA-seq

Antigen-specific B cells occur at very low frequencies (<1/100) in the peripheral blood B cell compartment. Given their low frequency, over the years many different methods have been developed to isolate and, in some cases, enrich for antigen-specific B cells from peripheral blood and tissues. All of these methods take advantage of the unique specificity of the B cell receptor (BCR) for a given antigen. Hence, in most cases identification and isolation of antigen-specific B cells entails baiting of the B cells of interest using the antigen they are specific for. For example, in our previous studies, we have isolated and enriched for insulin-reactive B cells in subjects with type 1 diabetes by incubating PBMCs with biotinylated insulin, followed by enrichment using anti-biotin magnetic beads and staining with a fluorescently labeled streptavidin (33, 34). While most often used with flow cytometry, identification of antigen-specific B cells can also be accomplished using mass cytometry, ELISPOT, and fluorescent microscopy (35–37). In late 2019 Ivelin Georgiev’s group expanded upon the available methods to study antigen-specific B cells to include scRNA-seq. In their ground breaking paper, they used biotinylated HIV antigens bound to fluorophore-conjugated streptavidin tetramers containing a unique oligonucleotide barcode to allow for downstream identification. Cells were exposed to the antigens, washed, and sorted by fluorescence activated cell sorting prior to single-cell capture. Their method, termed LIBRA-seq (linking B cell receptor to antigen specificity through sequencing), allows identification of antigen-specific B cells that can be multiplexed with transcriptomic and paired heavy and light chain BCR sequence data (24), greatly expanding the amount of information that can be learned from a single antigen-specific B cell in a high-throughput manner.

### Using scRNA-seq to study B cells during development of autoimmunity

While many have used scRNA-seq to study B cells in the context of infection, such as SARS-CoV-2, research teams have already begun applying scRNA-seq methods to the study of autoimmune diseases, though largely not focused on antigen-specific B cells. For example, Nehar-Belaid et al. identified a strong interferon stimulated gene (ISG) response among many immune cells, including B cells, but particularly in plasma cells, in children with SLE with high disease activity using scRNA-seq (38). More recently, scRNA-seq identified a disease relevant, CD180- B cell subset that is increased in MG patients and associated with increased disease activity and anti-AchR antibodies (39). As mentioned earlier, similar to findings using high dimensional flow and mass cytometry, scRNA-seq has confirmed expanded populations of Tbet+ ABCs in the synovial tissue from RA patients (40, 41) and the blood and kidney of SLE patients (38, 42). In addition, a recent study demonstrated the ability to integrate SLE-associated genetic variants with cell types and states identified using scRNA-seq in SLE patients (43). This study and others (44, 45) demonstrate the potential to not only link disease associated variants with their potential biological impact, but can also inform risk prediction and development of novel therapeutic strategies for precision-based medicine (46). Lastly, with the ability to combine gene expression data with BCR V(D)J sequences on a single cell level, studies have been able to identify clonal expansions of B cells sharing similar transcriptional phenotypes in the cerebral spinal fluid (CSF) of multiple sclerosis patients (29), and importantly, recently identified clonally expanded CSF-derived B cells that react to the central nervous system (CNS) protein GlialCAM and also cross-react with the Epstein-Barr virus (EBV) transcription factor, EBNA1 (47). Hence, while studies of B cells in autoimmune diseases using scRNA-seq are still in their infancy, the above examples highlight the potential of scRNA-seq to greatly expand our current knowledge regarding the phenotype of pathogenic B cells, their clonal expansion, cross-reactivity with viral antigens, and aptitude to traffic to target tissues.

## Discussion

**Figure 1:**
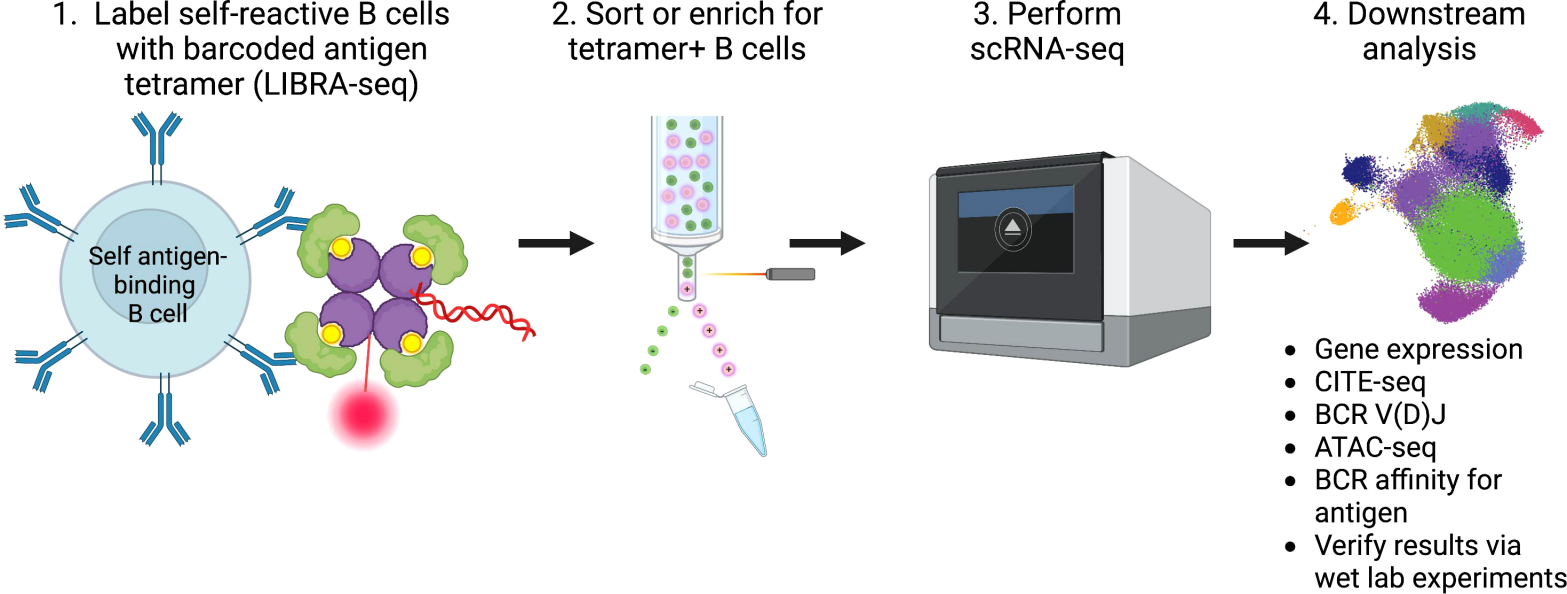
Summary of workflow and analysis of self-reactive B cells involved in the pathogenesis of autoimmunity using scRNA-seq.

Given the vast amount of knowledge that can be gained by studying the transcriptome and clonotype of B cells using scRNA-seq, we propose leveraging these technologies to help develop targeted treatment options for patients living with, or at increased risk for, autoimmune conditions, as well as help predict responses, or the lack thereof, to therapy. We envisage studies of B cells on an antigen-specific level, as the LIBRA-seq method employs, will yield the most informative results. Such studies entail creation of barcoded self-antigen reagents to identify self-reactive B cells from patients with autoimmune conditions, as well as controls. By combining antigen specificity, mRNA expression phenotypes, protein expression phenotypes using CITE-seq, epigenetic regulation changes using ATAC-seq, and BCR clonotypes through scRNA-seq, for example, insights gained from such analyses can help researchers calibrate potential therapeutics towards autoreactive B cells with unique expression profiles (**Figure 1**).

Moreover, the BCR sequences of self-reactive B cells identified within an individual or across autoimmune subjects can be queried for the identification of a “public” (i.e. shared) BCR V(D)J sequence among autoimmune subjects, similar to what has been found with some T cells in autoimmune diseases, including Celiac disease, T1D, and RA (48–51). Identification of shared BCRs among autoimmune subjects but not controls could then be therapeutically targeted through the use of anti-idiotypic antibodies (52), for example, which would spare non-pathogenic B cells. In addition, studies of BCR sequences in autoimmunity provide important information regarding the reactivity, specificity, and affinity of the offending B cells. By studying the germline and/or mutated BCR sequences of self-reactive B cells, one can predict how they were initially recruited into the autoimmune process, e.g. through germline self-reactivity, acquisition of somatic mutations in a germinal center reaction that conferred reactivity to self, or through cross-reactivity with a foreign antigen (53, 54). Such studies will inform our understanding of the interplay of B cells, the environment, and the pathogenesis of autoimmune diseases.

Despite the promise of using scRNA-seq to study the pathogenic B cells in autoimmunity, there are some limitations to the current method and analysis pipeline. The most commonly used platform, 10X Genomics, is limited by the number of cells that are recommended for input, which is around 10,000 for their standard immune profiling assay. More recently they have developed a High Throughput (HT) kit, yet the recommended number of cells tops out at 20,000 per lane for the immune profiling assay. Hence, in most cases enrichment and/or FACS sorting are necessary, which increases time *ex vivo* and can alter gene expression in the process. Additionally, when single cells are sequenced, the coverage of genes detected can sometimes be sparse and certain genes are known to be more difficult to detect by mRNA (e.g. CD8). Other limitations include: 1) the relatively few samples/subjects that can be processed on a single day compared to flow or mass cytometry, increasing the incidence of batch effects, 2) the high cost per sample, which has led to some studies publishing results from as few as one or two subjects, 3) the extensive time between day of sample processing and completion of data analysis, and 4) the need for collaborations with bioinformaticians to analyze the data who are in short supply. While scRNA-seq can provide more transcriptomic data on a single cell level than has been possible before, future technological advances are needed to allow for even more detailed high throughput analysis at a reasonable cost.

In addition, scRNA-seq technology and associated bioinformatic analysis required of the large datasets are relatively new methods for gathering data, even more so when you consider the study of antigen-specific B cells using this method. It is evident by the increasing number of papers critiquing some of the data analysis methods that biologists and informaticists are at odds with determining the most appropriate methods for normalization, batch correction, and statistical representation of the data (55–58). For example, it has been demonstrated that inclusion of the V(D)J transcripts in unsupervised clustering of B cells yields clusters that are based on V-gene segment usage and are not biologically meaningful clusters (59). Hence, it is better to remove V(D)J transcripts prior to unsupervised clustering. To this end, many analysis packages and tools are available open source for anyone to use which enables anyone to analyze their sequencing data and create plots representing their results. However, the largely non-reviewed aspect of this do-it-yourself approach by non-computational and informatics specialists could result in conclusions and reports that unintentionally misrepresent the actual data collected. There is a wide disparity in the amount of meaningful detail included per paper as to how figures and results were generated, making it difficult to repeat another group’s findings. Additionally, even informed bioinformaticians may not always fully consider or be able to represent the caveats when using one method over another or in applying an existing method to a slightly different type of sequencing experiment (57, 60), and it is, therefore, complicated to compare a study performed by one group to another. We should support more open sharing of data analysis methods and engage in thoughtful public dialogue about when certain methods are more or less appropriate to ensure accurate and reproducible data are being reported.

In relation to the analysis of antigen-specific B cells using scRNA-seq, there are also important considerations to take into account. Use of a bait sort approach, such as the LIBRA-seq method, makes it likely the antigen-tetramer will engage BCRs that are both low affinity and high affinity due to the increase in avidity generated by the tetramer itself. Low affinity antigen-specific B cells may actually be ignorant *in vivo*, not engaging in productive downstream BCR signaling and activation events, and therefore, should be excluded from the data analysis. Hence, it is important to determine which B cells are high affinity versus low affinity before making strong conclusions based on antigen-binding events. This may require traditional methods of determining BCR-antigen affinities by sequencing and cloning the BCR heavy and light chain sequences to make recombinant antibodies that can be tested for their affinity. Alternatively, the number of antigen counts per B cell may reflect a relative level of affinity. For example, a B cell that has 100 bound antigens may have a higher affinity than a B cell that only bound 5 antigens. Studies in our lab have indicated this is likely the case, but further studies from other groups are needed to verify or disprove this. In addition, if antigen tetramers are used, it is important to verify antigen-binding B cells are truly specific to the antigen and not another component of the tetramer, such as phycoerythrin (PE) or streptavidin. This can be accomplished through the use of “decoy” tetramers or dual labeling antigen-specific B cells (35, 61). Moreover, there is currently some discrepancy in the field as to what cut-off value of antigen-binding events equals a true antigen-specific B cell. Many studies do not report the threshold for calling a cell antigen-bound, and even further, the use of true negative or positive controls is not often reported. A more thoughtful discussion of the methods used and how cut-off values are decided is needed.

Another concern with any study of autoimmunity is that the timing of sample collection in the course of disease progression will be critical to develop a full understanding of how various subpopulations of immune cells contribute to different stages of disease. Researchers must be cognizant during data analysis that what is observed at one timepoint in disease onset or flare may differ from samples collected at a different time. Thus, longitudinal sampling and/or analysis at very early timepoints prior to treatment with immunosuppressive drugs will be crucial. Lastly, scRNA-seq conclusions are largely based on correlations, and therefore, require follow-up wet-lab experiments to verify true phenotypes exist, the biological mechanisms at play, as well as test novel therapeutics that are identified, in part, through the use of scRNA-seq. However, the use of scRNA-seq can aid in targeting those wet-lab experiments more efficiently by guiding researchers towards individual cell types and genes of interest that should be further studied in the context of autoimmune disease. Many groups have already demonstrated that combining scRNA-seq with wet-lab studies provides a powerful tool to advance our understanding of B cells in autoimmune diseases (46, 47, 62, 63). Once a convincing B cell phenotype or clonotype emerges, therapeutics can be developed to specifically target only the B cells contributing to disease, sparing healthy non-pathogenic B cells. In conclusion, we anticipate that harnessing the methods and vast amount of knowledge gained through use of scRNA-seq technologies, while being cognizant of its limitations, will help further our understanding of the role of B cells in development of autoimmunity and identify potential therapeutic targets.

## Data availability statement

The original contributions presented in the study are included in the article/supplementary material. Further inquiries can be directed to the corresponding author.

## Author contributions

CN and MS conceptualized, wrote, edited, and reviewed this manuscript. Funding was provided by MS. All authors contributed to the article and approved the submitted version.
